# Subtraction iodine imaging with area detector CT to improve tumor delineation and measurability of tumor size and depth of invasion in tongue squamous cell carcinoma

**DOI:** 10.1007/s11604-021-01196-4

**Published:** 2021-09-16

**Authors:** Takashi Hiyama, Hirofumi Kuno, Kotaro Sekiya, So Tsushima, Shioto Oda, Tatsushi Kobayashi

**Affiliations:** 1 Department of Diagnostic Radiology, National Cancer Center Hospital East, 6-5-1, Kashiwanoha, Kashiwa, Chiba 277-8577 Japan; 2 Canon Medical Systems Corporation, Otawara, Tochigi Japan

**Keywords:** Subtraction, Computed tomography, Tongue, Squamous cell carcinoma, Artifact

## Abstract

**Purpose:**

Tumor size and depth of invasion (DOI) are mandatory assessments for tumor classification in tongue cancer but are often non-assessable on CT due to dental artifacts. This study investigated whether subtraction iodine imaging (SII) would improve tumor delineation and measurability.

**Materials and methods:**

Fifty-seven consecutive patients with tongue cancer, who underwent scanning with a 320-row area detector CT with contrast administration and were treated with surgical resection, were retrospectively evaluated. CT was reconstructed with single-energy projection-based metallic artifact reduction (sCT). SII was generated by subtracting the pre-contrast volume scans from the post-contrast volume scans using a high-resolution deformable registration algorithm. MRI scans were also evaluated for comparing the ability of measurements. Two radiologists visually graded the tumor delineation using a 5-point scale. Tumor size and DOI were measured wherever possible. The tumor delineation score was compared using the Wilcoxon signed-rank method. Spearman’s correlations between imaging and pathological measurements were calculated. Intraclass correlation coefficients of measurements between readers were estimated.

**Results:**

The tumor delineation score was greater on sCT-plus-SII than on sCT alone (medians: 3 and 1, respectively; *p* < 0.001), with higher number of detectable cases observed with sCT-plus-SII (36/57 [63.2%]) than sCT alone (21/57 [36.8%]). Tumor size and DOI measurability were higher with sCT-plus-SII (29/57 [50.9%]) than with sCT alone (17/57 [29.8%]). MRI had the highest detectability (52/57 [91.2%]) and measurability (46/57 [80.7%]). Correlation coefficients between radiological and pathological tumor size and DOI were similar for sCT (0.83–0.88), sCT-plus-SII (0.78–0.84), and MRI (0.78–0.90). Intraclass correlation coefficients were higher than 0.95 for each modality.

**Conclusions:**

SII improves detectability and measurability of tumor size and DOI in patients with oral tongue squamous cell carcinoma, thus increasing the diagnostic potential. SII may also be beneficial for cases unevaluable on MRI due to artifacts or for patients with contraindications to MRI.

## Introduction

Oral tongue squamous cell carcinoma (OTSCC) is the most common carcinoma among oral cavity cancers [1]. Tumor size and depth of invasion (DOI) are mandatory for tumor classification in OTSCC according to the American Joint Committee on Cancer (AJCC) eighth edition [2]. Pathological DOI is associated with nodal metastasis and is a negative prognostic factor [3]. Accurate measurement of tumor size and DOI by cross-sectional imaging is therefore essential for predicting the prognosis, determining the tumor classification, and determining optimal treatment strategies.

CT is widely used as the basic imaging modality in the preoperative evaluation of patients with OTSCC. Previous studies have demonstrated the utility of CT for primary tumors, such as in cases of invasion of the neurovascular bundle, extrinsic muscular invasion, mandibular invasion, and DOI measurement [4–8]. Furthermore, CT allows for the assessment of distant metastases in addition to primary tumors and nodal metastases. However, the primary tumor is often non-assessable on CT due to dental artifacts. The metallic artifact reduction (MAR) algorithm is an effective artifact reduction technique for CT [9–17]. Although it is well-established that the MAR algorithm improves the imaging quality of the oral cavity [11–17], there are numerous cases in which tongue cancer cannot be clinically delineated despite the use of MAR.

Subtraction iodine imaging (SII) is employed in several musculoskeletal and neurological applications [18–21]. SII is generated by subtracting the pre-contrast CT from the post-contrast enhanced CT. This technique reduces spatial mismatch using volume scanning with a 160-mm wide area detector CT and a high-resolution deformable registration algorithm, enabling identification of contrast enhancement. SII has demonstrated good performance for bone marrow lesions due to accentuation of iodine distribution. As SII accentuates contrast enhancement and has the potential to reduce artifacts by subtracting artifacts, we hypothesized that SII would improve OTSCC delineation and measurability. The aim of this study was thus to evaluate whether the addition of SII to standard CT with the MAR algorithm would improve tumor delineation and measurability of tumor size and DOI in patients with tongue cancer. We also evaluated MRI scans for comparing the ability of measurements, which have a better soft-tissue resolution and are used to assess the local extension of OTSCC. Further, the radiological and pathological measurements of tumor size and DOI were investigated on each modality and correlated.

## Materials and methods

### Study population

**Fig. 1:**
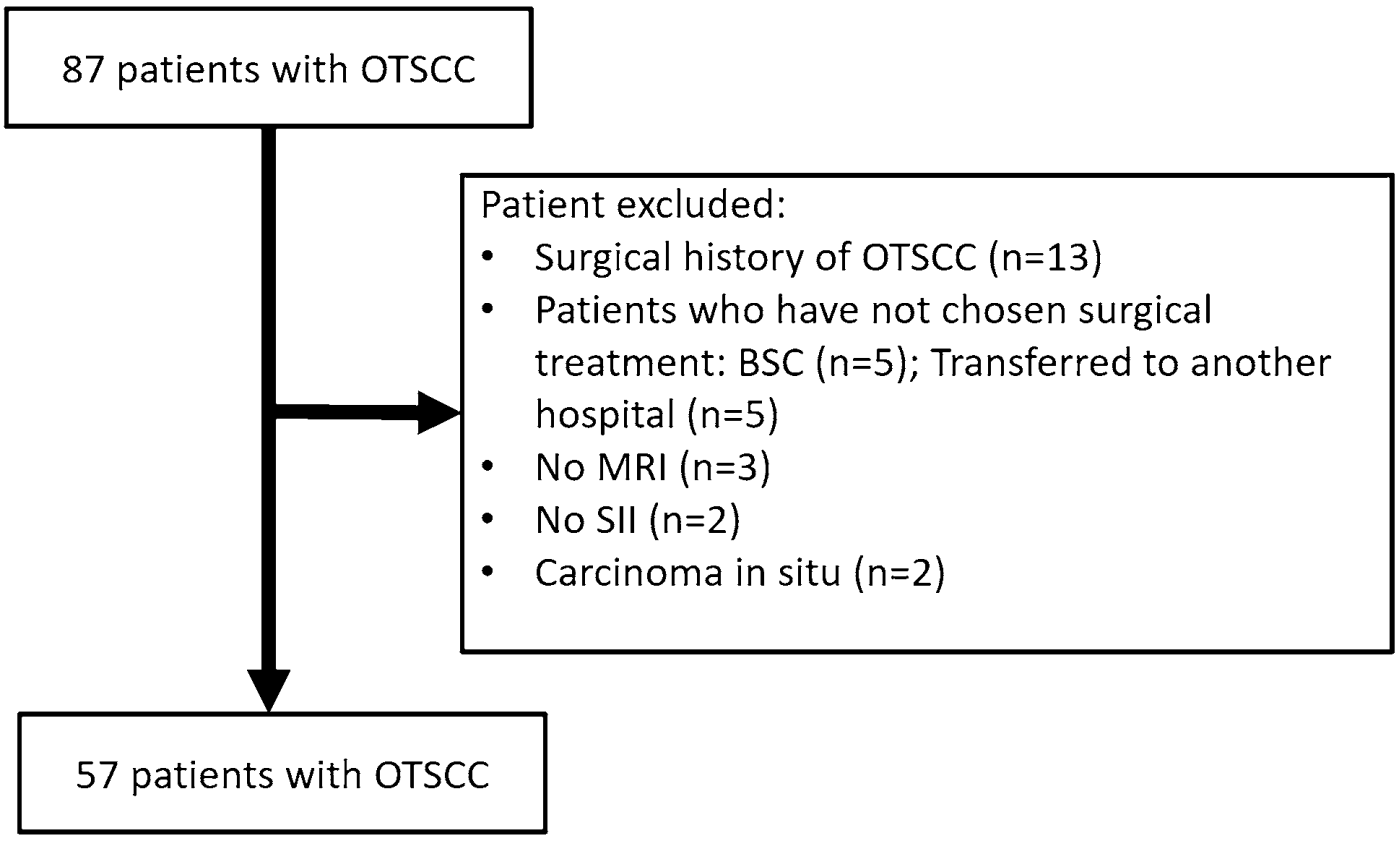
Flowchart of patient selection. *OTSCC* oral tongue squamous cell carcinoma, *BSC* best supportive care, *SII* subtraction iodine imaging

Our institutional review board at (National Cancer Center Hospital East for review) approved this retrospective study. The requirement for written informed consent was waived. Eighty-seven consecutive newly diagnosed patients with histologically proved OTSCC underwent contrast-enhanced CT for pre-treatment cancer staging between January 2018 and August 2019. Eighty-four of these patients underwent contrast-enhanced 3-T MRI before the treatment. Among the 84 patients, 13 were excluded as they had undergone operation for oral cancer while 10 patients were excluded due to non-surgical treatment such as best supportive care or transfer to other hospitals. Further, two patients were excluded due to unavailable volume data required for subtraction reconstruction, and two patients with carcinoma in situ were excluded due to non-measurability of pathological DOI. The remaining 57 patients were considered (Fig. 1).

### Subtraction iodine imaging techniques

All CT images were obtained using a 320-row detector CT system with a 160-mm detector width (Aquilion ONE Vision; Canon Medical Systems) and the following parameters: 120 effective mAs, 120 kV, 0.5 s rotation time, and 160-mm collimation. The average CT dose index was 25.2 mGy. Nonionic contrast media (80 mL of iopamidol 300 mgI/mL (Bystage; Teva Takeda Pharma) for seven patients with a body weight of < 45 kg, 100 mL of iopamidol 300 mgI/mL (Bystage) for 47 patients with a body weight of 45–75 kg, or 100 mL of iopamidol 370 mgI/mL (Oypalomin; Fuji Pharma) for three patients with a body weight of > 75 kg) were administered at 2.5 mL/s into the antecubital vein through a 22-G cannula. Scans at 7 s (mask volume) and 70 s (post-contrast volume) after starting the injection were obtained.

The two volume datasets (7- and 70-s CTs) were reconstructed using a single-energy projection-based MAR algorithm (SEMAR; Canon Medical Systems) with three-dimensional adaptive iterative dose reduction (AIDR 3D; Canon Medical Systems). Mask volume was subtracted from post-contrast volume using the SURE Subtraction application with a high-resolution deformable registration algorithm (^SURE^Subtraction Neck, Canon Medical Systems). Axial and coronal CT with a single-energy projection-based MAR algorithm (sCT) with a soft-tissue window (window level: 60, window width: 350) and axial and coronal SII (window level: 50, window width: 130) were reconstructed. sCT and SII were reconstructed to yield precisely matching slices (1-mm slice thickness, 512 × 512 matrix, and 16-cm field of view).

### MRI protocol and parameters

MRI was performed with a 3.0-T scanner (Achieva or Ingenia; Philips Healthcare). Axial T1-weighted images (repetition time (TR)/echo time (TE), 675/13; 560 × 560 matrix; 24-cm field of view; slice thickness, 3.0 mm; flip angle, 90°; sensitivity encoding (SENSE) factor, 2.5), axial and coronal T2-weighted images (TR/TE, 4868/90; 560 × 560 matrix; 24-cm field of view; slice thickness, 3.0 mm; flip angle, 90°; SENSE factor, 2.5), and coronal short inversion time inversion-recovery images (TR/TE/inversion time (TI), 4560/60/220; 512 × 512 matrix; 24-cm field of view; slice thickness, 3.0 mm; flip angle, 90°; SENSE factor, 2.5) were obtained using a 16-channel head-and-neck coil. After administration of 0.1 mmol of gadobutrol (Gadovist; Bayer) or gadopentetate (Magnevist; Bayer) per kg of body weight, axial three-dimensional T1-weighted Dixon gradient echo water images (TR/first TE/second TE, 4/1.5/2.5; 320 × 320 matrix; slice thickness, 1.0 mm; flip angle, 15°; SENSE factor, 2.5) were obtained. Coronal post-contrast T1-weighted images (section thickness of 1.2 mm with 1.2-mm intersection gap) were reconstructed from three-dimensional sequences.

### Image interpretation

Two radiologists (with 14 (H.K.) and 13 years (T.H.) of experience in oncologic diagnostic radiology) independently analyzed the images. Readers were only informed of the OTSCC subsites (left or right tongue border, or underside of the tongue) and were National Cancer Center Hospital East to patients’ clinical histories and images from other modalities.

#### Artifact evaluation for sCT and SII

Readers evaluated artifacts on sCT and SII using a 5-point scale: 1, severe artifacts, largely not diagnostic; 2, poor image quality, partly non-diagnostic; 3, moderate image quality, limited diagnostic confidence; 4, good image quality, sufficient for diagnosis; 5, excellent image quality, no artifacts. For quantitative analysis, one reader measured image noise as the circular region-of-interest standard deviation in the oral cavity. The circular region-of-interest was approximately 400 mm^2^ and was placed on the tongue on the slice containing the strongest artifact. The circular region-of-interest was placed at the same location on sCT and SII slices using the copy-paste function. To determine the contrast-to-noise ratio (CNR), the averages of the Hounsfield units (HU) of tumor (HU_tumor_) and tongue (HU_tongue_) were measured in detectable cases both on sCT and SII. The CNR was calculated as follows: (HU_tumor_ − HU_tongue_)/(standard deviation of HU_tongue_). 

#### Evaluation of tumor delineation and detectability

Two National Cancer Center Hospital East readers visually graded the tumor delineation score using a 5-point scale as follows: 5, the whole tumor was visible; 4, most of the tumor was visible; 3, the tumor was partially visible; 2, uncertain of tumor presence; 1, the tumor was definitely nonvisible. Scores of 3–5 were defined as detectable.

#### Radiological tumor size and DOI measurements

Tumor size and DOI were measured wherever possible on each modality. Widest dimensions on axial or coronal images were considered for tumor size. As to MRI, tumor size and DOI were measured on contrast-enhanced T1-weighted MR images with fat suppression. DOI was measured by drawing a perpendicular line from the reference line connecting the junction of both side points of the tumor and normal mucosa to the tumor’s deepest point.

sCT, sCT-plus-SII, and MRI assessments were each performed at least 4 weeks apart. Images were randomly presented in three sessions using a workstation (Shade Quest View R; Yokogawa Electric). The final score was determined by consensus between the readers.

### Histopathological analysis

Tissue samples were fixed in formalin, embedded, sectioned, and stained with hematoxylin and eosin. Pathological DOI was measured according to AJCC by identifying the "horizon'' of the adjacent squamous mucosa basement membrane. A perpendicular plumb line was established from this horizon to the deepest point of tumor invasion, which represented the DOI. Pathological tumor size and DOI measurements were obtained from histopathology reports.

### Statistical analysis

Artifact and tumor delineation scores were compared using the Wilcoxon signed-rank method. Bonferroni correction was applied for multiple comparisons. Inter-reader agreement between readers’ independent evaluations of artifact and tumor delineation scores for each modality were estimated with weighted kappa statistics (weight = 2). A paired t-test was used to compare image noise and CNR. The frequencies of detectable or measurable cases on each modality were compared using the *χ*^2^ test with Bonferroni correction. The Shapiro–Wilk test showed that tumor size and DOI were not normally distributed. Therefore, Spearman’s correlations were used to evaluate the correlation between imaging and pathological measurements for tumor size and DOI. These correlations were evaluated between measurements on sCT or SII and those on MRI. Intraclass correlation coefficients (ICC) were estimated to evaluate the degree of absolute agreement on tumor size and DOI measurements between readers for each modality using a two-way random effects model and single rating. The ICC interpretation was as follows: < 0.5, poor; 0.5–0.75, moderate; 0.75–0.9, good; > 0.9, excellent [22]. Commercial software (STATA, Version 12.1; StataCorp) was used for statistical analyses. *p* < 0.05 was considered statistically significant.

## Results

### Patient demographics

We considered 57 patients comprising 35 men and 22 women (age range, 30–89 years; mean age, 61.5 years; standard deviation, 15.8 years). Tumor stages T1, T2, T3, and T4a were noted in 14, 23, 14, and 6 cases, respectively, according to the 8th edition of the AJCC. Metallic prosthetic appliances were observed in 48 cases (5 unilateral and 43 bilateral cases). The average duration between CT imaging and surgery was 20.8 days (range 8–66, median 28), and between MRI and surgery was 20.1 days (range 7–37, median 20). The mean estimated glomerular filtration rate (eGFR) was 76.4 mL/min/1.73 m^2^ (standard deviation 19.9). The detailed clinical profiles are summarized in Table 1.

**Table 1:** Summary of patient characteristics

Demographic	Value (*n* = 57)
Age (years)	
Mean (standard deviation)	61.5 (15.8)
Range	30–89
Sex^a^	
Male	35 (61.4)
Female	22 (38.6)
Clinical T classification^a,b^	
T1	14 (24.6)
T2	23 (40.3)
T3	14 (24.6)
T4a	6 (10.5)
T4b	0 (0)
Clinical N classification^a,b^	
N0	41 (71.8)
N1	7 (12.3)
N2a	0 (0)
N2b	3 (5.3)
N2c	3 (5.3)
N3a	0 (0)
N3b	3 (5.3)
Clinical M classification^a,b^	
M0	57 (100)
M1	0 (0)
Subsites^a^	
Left tongue border	31 (54.3)
Right tongue border	23 (40.4)
Underside of the tongue	3 (5.3)
Metallic prosthetic appliance^a^	
None	9 (15.8)
Unilateral	5 (8.8)
Bilateral	43 (75.4)
Surgical procedure^a^	
Partial glossectomy	38 (66.7)
Subtotal glossectomy	10 (17.5)
Hemiglossectomy	7 (12.3)
Total glossectomy with total laryngectomy	2 (3.5)

^a^Denotes the number of patients within each category. Numbers in parentheses represent the percentage within a given group

^b^Clinical TNM classification according to the eighth edition of the American Joint Committee on Cancer staging system

### Artifact score and imaging noise

**Fig. 2:**
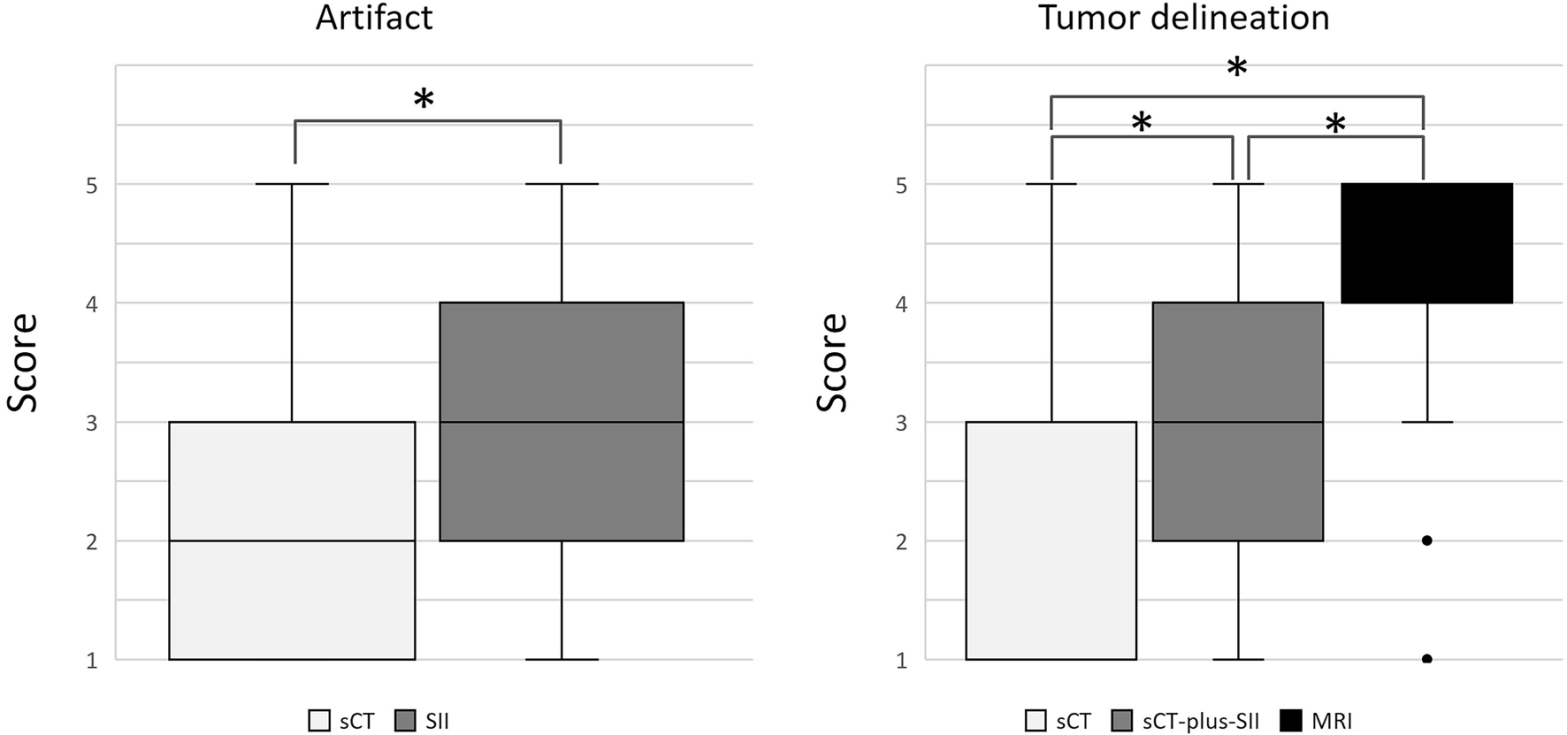
The box and whisker plot shows artifact scores (**A**) and tumor delineation scores (**B**). Boxes correspond to 25th and 75th percentiles for the scores in each modality. Whiskers denote maximum and minimum values. Lines in the box represent the median. Median values of sCT and MRI in tumor delineation are 1 and 4, respectively. *p < 0.001, *sCT* CT with single-energy projection-based metallic artifact reduction algorithm, *SII* subtraction iodine imaging

The median (interquartile range) final evaluation artifact scores were 2 (1–3) and 3 (2–4), for sCT and SII, respectively (Fig. 2); this difference was statistically significant (*p* < 0.001). Inter-reader agreement of sCT and SII artifact scores was 0.76 and 0.70, respectively. There were five cases with worse artifacts on SII than on sCT, with a difference in artifact scores of 1 or 2. In these cases, artifacts were caused by subtraction mismatching of dental artifacts. Other cases had similar or better scores on SII and sCT. There was a significant difference in mean image noise between sCT and SII (48.3 ± 27.8 HU and 28.2 ± 9.63 HU, respectively; *p* < 0.0001). Although the CNR was slightly higher on SII than on sCT, there was no significant difference in CNR between sCT and SII (2.45 ± 1.15 and 2.70 ± 1.26, respectively; *p* = 0.31).

### Tumor delineation score and detectability

**Fig. 3:**
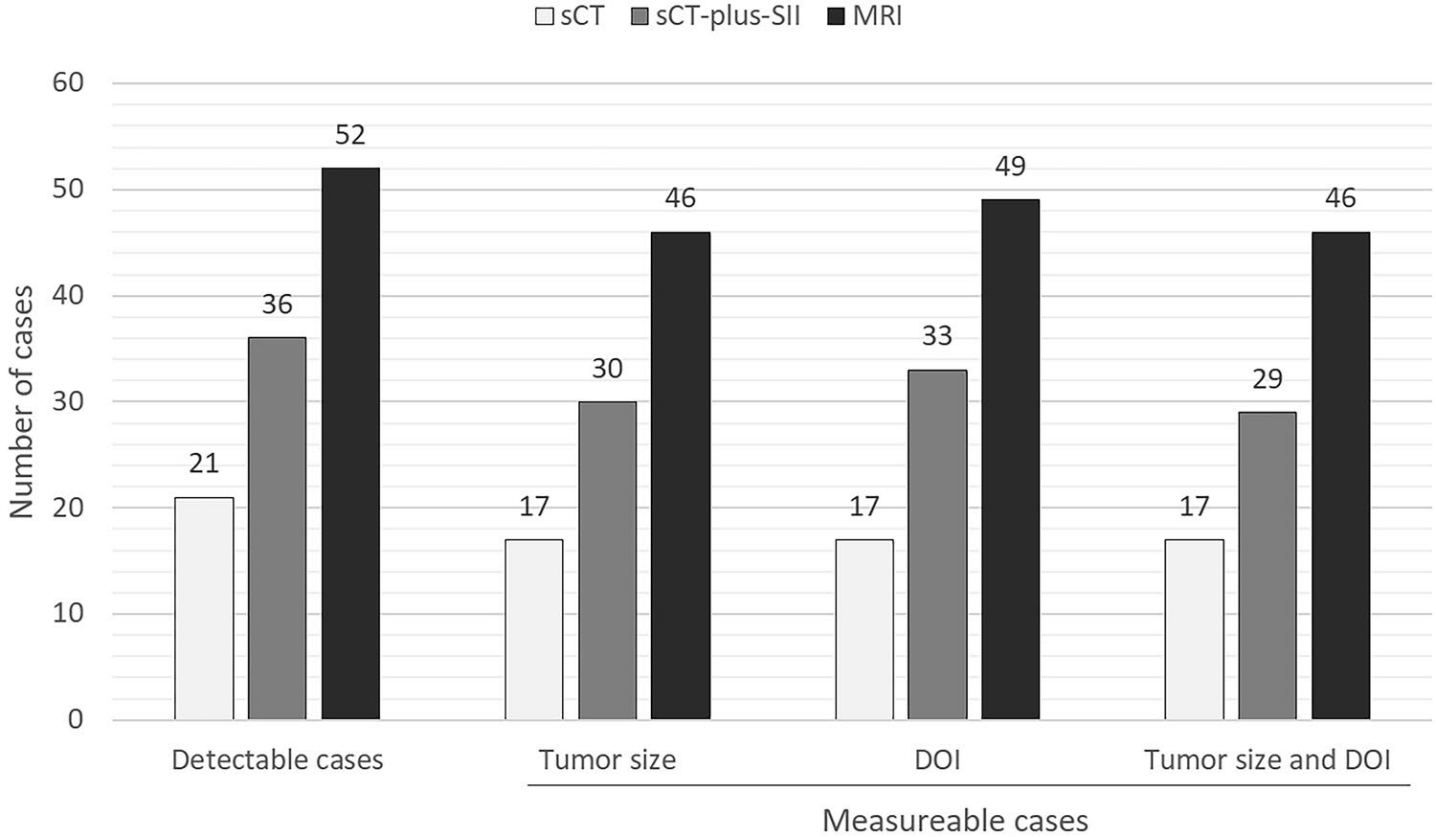
Bar chart shows detectable and measurable cases for each modality. The number represents the cases evaluable by two raters. Measurable cases include those for tumor size, depth of invasion (DOI), and both tumor size and DOI. *sCT* CT with single-energy projection-based metallic artifact reduction algorithm, *SII* subtraction iodine imaging

**Fig. 4:**
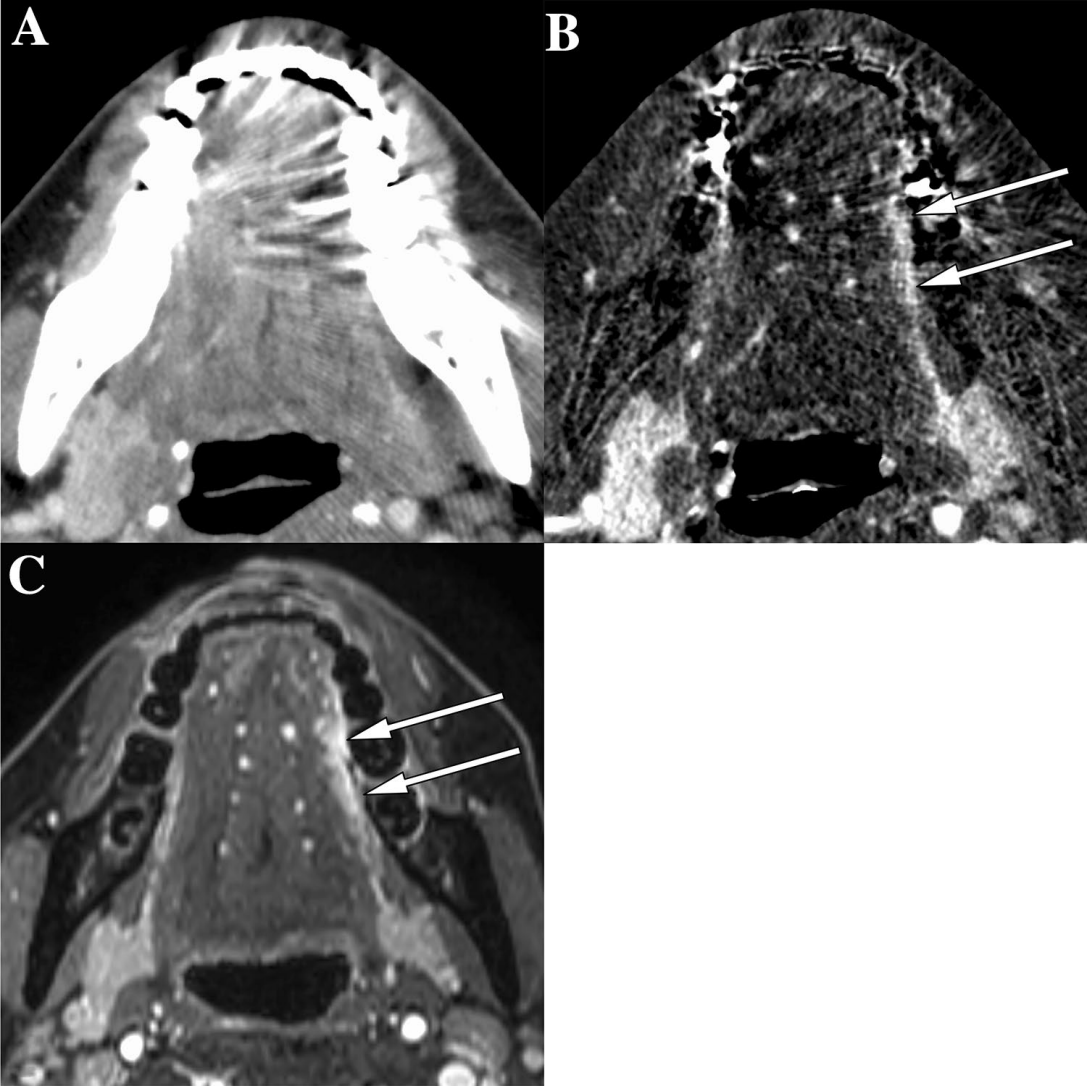
Images of a 48-year-old woman with squamous cell carcinoma of the left border of the tongue. **A** On CT with single-energy projection-based metallic artifact reduction algorithm, tongue cancer was not detectable due to dental artifacts. **B** Subtraction iodine imaging (SII) shows tumor enhancement of the left border of the tongue (white arrows in **B**) mainly due to reduced artifacts using subtraction. **C** On contrast-enhanced T1-weighted MR images with fat suppression, the tumor is detectable and measurable (white arrows in **C**) as well as with SII

**Fig. 5:**
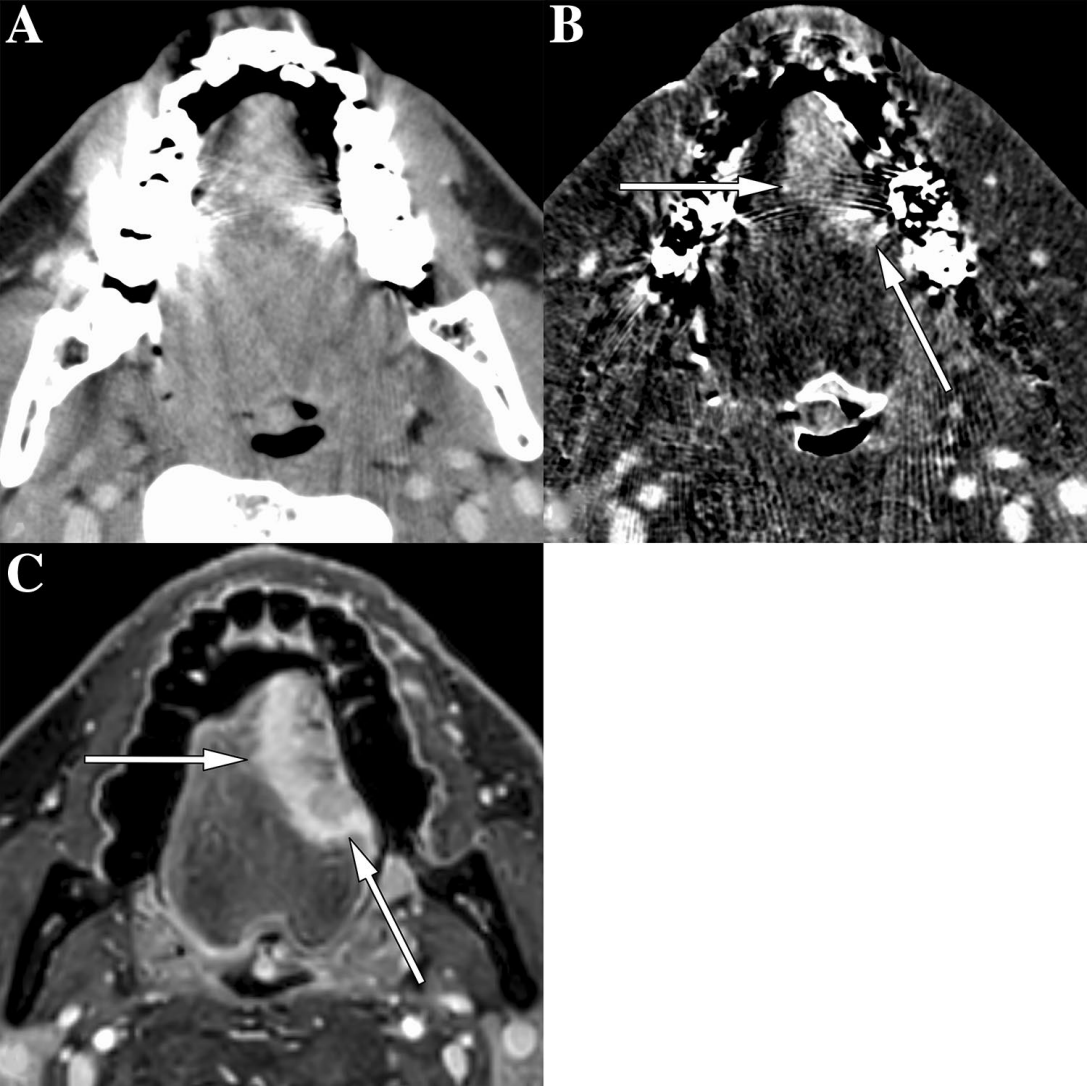
Images of a 43-year-old man with squamous cell carcinoma of the left border of the tongue. **A** On CT with single-energy projection-based metallic artifact reduction algorithm, tongue cancer was not detectable due to bilateral dental artifacts. **B** Subtraction iodine imaging (SII) is able to delineate the tumor mainly due to accentuation of iodine and increasing contrast between the tumor and tongue muscles. **C** On contrast-enhanced T1-weighted MR images with fat suppression, the tumor is detectable and measurable (white arrows in **C**) as well as with SII

The median (interquartile range) tumor delineation scores were 1 (1–3), 3 (2–4), and 4 (4–5) for sCT, sCT-plus-SII, and MRI, respectively (Fig. 2). The Wilcoxon signed-rank method with Bonferroni correction revealed a significant difference among modalities (*p* < 0.001). The inter-reader agreements of the delineation score on sCT, sCT-plus-SII, and MRI were 0.93, 0.81, and 0.74, respectively. Detectable cases (tumor delineation scores = 3, 4, 5) of sCT-plus-SII images were superior to those of sCT (36/57 cases [63.2%] vs. 21/57 [36.8%]), although MRI had the highest detectability (52/57 cases [91.2%]) (Fig. 3). The statistical evaluation of the frequencies of detectable cases revealed a significant difference among modalities (*p* < 0.05). All cases detectable on sCT were detectable on sCT-plus-SII. Of the 15 cases identifiable on sCT-plus-SII but not sCT, seven were detectable due to reduction in artifacts (Fig. 4). Four cases were detectable due to accentuation of tumor contrast enhancement leading to increased contrast between the tumor and normal lingual tissue (Fig. 5), while the other four were rendered detectable due to the relatively thin lesions on the tongue edge and greater contrast enhancement. All cases detectable on sCT-plus-SII were detectable on MRI.

### Radiological tumor size and DOI measurability

**Fig. 6:**
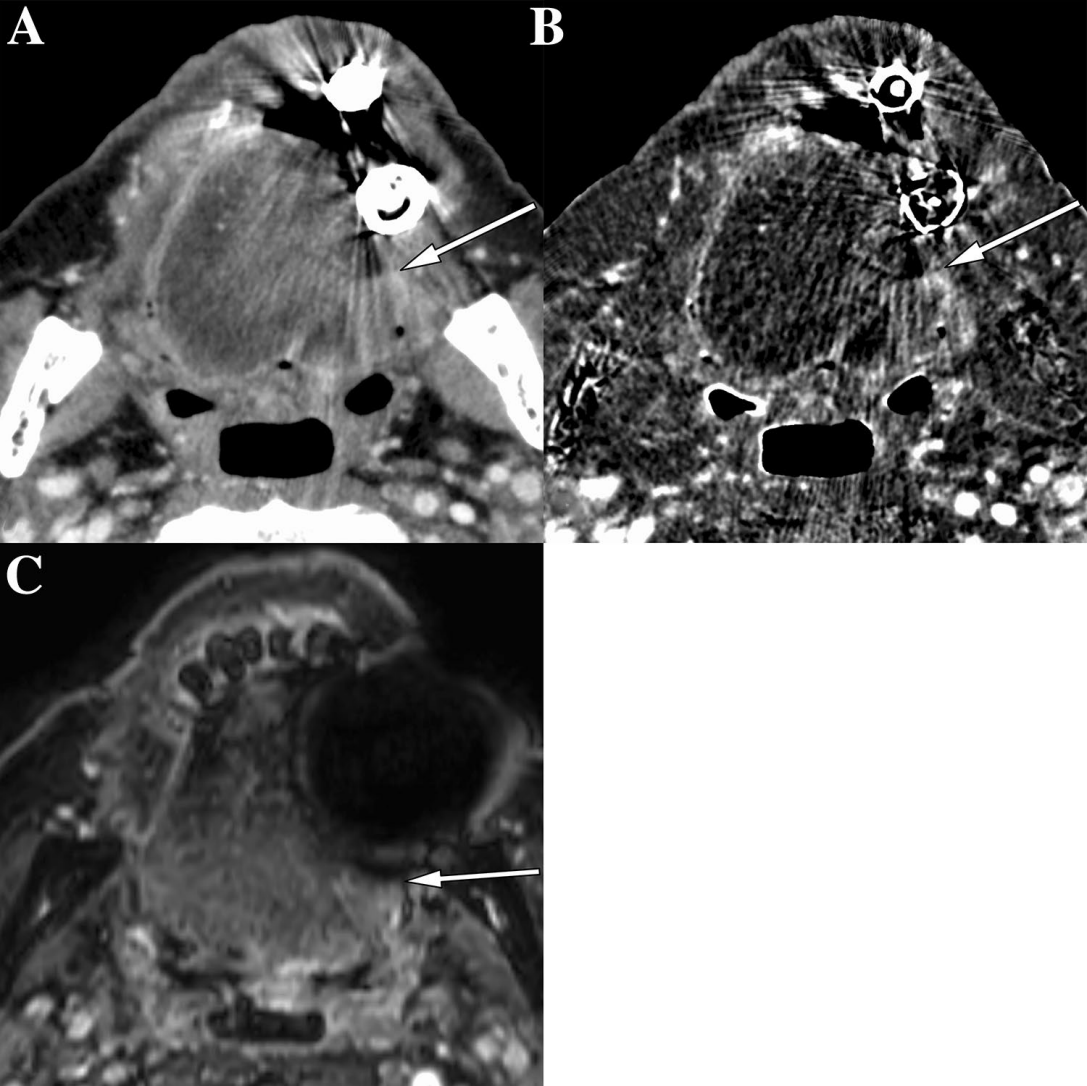
Images of a 74-year-old man with squamous cell carcinoma of the left border of the tongue. **A**, **B** On CT with the single-energy projection-based metallic artifact reduction algorithm (**A**) and subtraction iodine imaging (**B**), the anterior part of the tumor is delineated (arrows in **A** and **B**), and the tumor size is measurable. **C** On contrast-enhanced T1-weighted MR images with fat suppression, the anterior part of a tumor cannot be delineated due to dental artifacts, and the tumor size is unmeasurable

The numbers of measurable tumor size reported by both readers were 17/57 (29.8%) sCT cases, 30/57 (52.6%) sCT-plus-SII cases, and 46/57 (80.7%) MRI cases (Fig. 3). The frequencies of cases with measurable tumor size were significantly different between sCT or sCT-plus-SII and MRI (both *p* < 0.01), but not between sCT and sCT-plus-SII (*p* = 0.396). All cases measurable on sCT were measurable on sCT-plus-SII. One case unmeasurable on MRI due to strong dental artifacts was measurable on both sCT and sCT-plus-SII imaging (Fig. 6). The other case unmeasurable on MRI due to motion artifacts was measurable on sCT-plus-SII only. A total of 18 cases measurable on MRI were unmeasurable on both sCT and sCT-plus-SII due to undetectability or dental artifacts.

The numbers of measurable DOIs reported by both readers were 17/57 (29.8%) sCT cases, 33/57 (57.9%) sCT-plus-SII cases, and 49/57 (86.0%) MRI cases (Fig. 3); these frequencies were significantly different among modalities (*p* < 0.05). All cases measurable on sCT were measurable on sCT-plus-SII. One case unmeasurable on MRI due to motion artifacts was measurable on sCT-plus-SII imaging only. A total of 17 cases measurable on MRI were unmeasurable on both sCT and sCT-plus-SII due to undetectability or dental artifacts.

The numbers of measurable cases for both tumor size and DOI were 17/57 (29.8%) sCT cases, 29/57 (50.9%) sCT-plus-SII cases, and 46/57 (80.7%) MRI cases (Fig. 3). The statistical evaluation of these frequencies revealed a significant difference between sCT or sCT-plus-SII and MRI (both *p* < 0.01), but not between sCT and sCT-plus-SII (*p* = 0.07).

### Correlations between radiological and pathological measurements

The results of the correlation analysis of radiological and pathological measurements for each reader are summarized in Table 2. The correlation coefficients between radiological and pathological measurements were 0.83–0.88, 0.78–0.84, and 0.78–0.90 for sCT, sCT-plus-SII, and MRI, respectively. The correlations of sCT and sCT-plus-SII measurements with MRI measurements are summarized in Table 3. The correlation coefficients were 0.85–0.94 and 0.80–0.96 for sCT and sCT-plus-SII, respectively. The ICCs between readers for tumor size and DOI were higher than 0.95 for each modality and were considered excellent (Table 4).

**Table 2:** Spearman correlation coefficients between radiological and pathological measurements for each rater

Modality	Tumor size	Depth of invasion
Rater 1		
sCT	0.86 (21)	0.83 (20)
sCT-plus-SII	0.78 (36)	0.84 (35)
MRI	0.78 (52)	0.84 (53)
Rater 2		
sCT	0.84 (18)	0.88 (18)
sCT-plus-SII	0.83 (31)	0.84 (35)
MRI	0.82 (46)	0.90 (49)

Numbers in parentheses represent the number of measurable cases for each rater among 57 patients

*sCT* CT with the single-energy projection-based metallic artifact reduction algorithm, *SII* subtraction iodine imaging

**Table 3:** Spearman correlation coefficients between measurements on CT/sCT-plus-SII and those on MRI for each rater

Modality	Tumor size	Depth of invasion
Rater 1		
sCT-plus-SII	0.85 (35)	0.80 (33)
sCT	0.94 (20)	0.86 (20)
Rater 2		
sCT-plus-SII	0.89 (28)	0.88 (33)
sCT	0.88 (20)	0.96 (18)

Numbers in parentheses represent the number of measurable cases for each rater among 57 patients

*sCT* CT with the single-energy projection-based metallic artifact reduction algorithm, *SII* subtraction iodine imaging

**Table 4:** Intraclass correlation coefficients for each modality

Modality	Tumor size	Depth of invasion
sCT	0.96 (17)	0.97 (17)
sCT-plus-SII	0.97 (30)	0.98 (33)
MRI	0.97 (46)	0.98 (49)

Numbers in parentheses represent the number of measurable cases for both raters among 57 patients

*sCT* CT with the single-energy projection-based metallic artifact reduction algorithm, *SII* subtraction iodine imaging

## Discussion

Tumor size and DOI assessments are mandatory for tumor classification in OTSCC according to the 8th edition of the AJCC guidelines [2]. However, dental artifacts often preclude the ability of CT to assess these parameters, even with the application of the MAR algorithm. Here, we demonstrated that adding SII to CT with the MAR algorithm increased the number of detectable cases from 21/57 (36.8%) to 36/57 (63.2%) and the number of measurable cases of both tumor size and DOI from 17/57 (29.8%) to 29/57 (50.9%), although MRI had the highest detectability 52/57 (91.2%) and measurability 46/57 (80.7%). SII may be beneficial for cases that cannot be evaluated with MRI due to large metal or motion artifacts or for patients with contraindications to MRI due to intracranial or orbital metallic foreign bodies, MRI-unsafe pacemaker devices, or cochlear implants.

The MAR algorithm, which replaces corrupted projections by interpolation from uncorrupted projections, is effective for reducing artifacts due to photon starvation causing pronounced metal artifacts [9]. Several studies have reported that CT with the MAR algorithm improved the imaging quality and detectability of lesions in the oral region [11–17]. Previous studies have indicated that CT with the MAR algorithm was able to detect 22–56% more tumors compared with conventional CT [12, 13]. However, tumor measurability has not been assessed. Fewer OTSCCs were detected (even on sCT) in our study than in previous studies, and there were fewer measurable cases than detectable cases.

SII has been used for visualizing contrast enhancement in bone [18–21, 23]. One study reported that the extent of bone marrow infiltration by nasopharyngeal carcinoma was visualized on SII by accentuating contrast enhancement and suppressing normal bony tissues [19]. In the present study, SII improved tumor delineation in soft tissues by accentuating contrast enhancement. Further, SII exhibited the potential to obscure dental artifacts by subtracting the artifacts, thus improving OTSCC visualization. Several studies have demonstrated that dual-energy CT reduced beam-hardening artifacts using virtual monochromatic images at high kilo-electron volt levels and allowed combined usage with the MAR algorithm [24–26]. However, there is a trade-off between reduced tissue contrast and images obtained with high kilo-electron volt levels [27, 28]. In this regard, SII may compensate for weakened tumor contrast on virtual monochromatic images at high kilo-electron volt levels. Further, Baerends et al. showed in a phantom study that sCT has a higher contrast-to-noise ratio and reduced iodine discrimination thresholds compared with dual-energy CT [29].

DOI is well-established as a negative prognostic factor and is included in the tumor classification criteria [2, 3]. DOI derived from MRI/CT and pathological DOI are strongly correlated [5, 30–32]. Baba et al. reported that CT-derived DOI exhibited a stronger correlation and better approximation with pathological DOI when compared to MRI-derived DOI [5]. However, 84.9% of cases are non-assessable by CT due to technical limitations including dental artifacts. Reports on the reproducibility of the radiological DOI indicate that inter-reader agreement for MRI is good [33, 34]. In this study, DOI and tumor size measured on sCT-plus-SII images correlated well with pathological measurements, similar to sCT and MRI. In addition, inter-reader agreement for all imaging modalities was excellent. Therefore, sCT-plus-SII may have sufficient quality for clinical usage.

There are several limitations to our study. First, several cases presented with worsened artifacts on SII due to a mismatch of subtraction and enhancement of artifacts represented on sCT. Reducing spatial mismatch and developing methods to generate the same artifacts in pre- and post-contrast CT may resolve this problem. Second, even on sCT-plus-SII, 49.1% of cases were unmeasurable. Artifact reduction using SII renders artifacts less noticeable but does not visualize contrast enhancement of the tumor itself. Although the suppression of artifacts improved tumor visibility in our study, improvements in artifact reduction techniques such as the MAR algorithm or dual-energy CT are necessary to reduce radical artifacts. Third, we did not consider the shrinkage factor of fixation when comparing radiological/pathological measurements. It is well-established that the radiological DOI is larger than the pathological DOI because specimens shrink when fixed or if inflammation occurs [1, 5, 31–34]. Furthermore, tumor sizes may change between CT, MRI, and surgery. Since the main purpose of the present study was to evaluate the detectability and measurability of sCT-plus-SII, we did not take the shrinkage factor into consideration. Prospective studies are required to coordinate the duration of image acquisition and surgery. Fourth, we used gradient-echo sequences for contrast-enhanced T1-weighted imaging. Because magnetic susceptibility artifacts are more pronounced on gradient-echo sequences, spin-echo sequences may improve detectability and measurability on MRI. Finally, we have not evaluated how differences in modality influence the T classification; the current tongue cancer staging system includes not only size and DOI but also mandibular and masticator invasion. The impact of SII on T classification needs to be assessed in future studies.

## Conclusions

SII in combination with sCT improves the detectability and measurability of tumor size and DOI in patients with OTSCC and increases the number of diagnosable tumor classifications. Correlations between radiological and pathological measurements and inter-rater agreement were similar among sCT, sCT-plus-SII, and MRI. SII may increase the diagnostic potential in the patient with OTSCC and may also be beneficial for cases unevaluable on MRI due to dental or motion artifacts or for patients with contraindications to MRI.
